# Outcomes of Injection Laryngoplasty for Deep Interarytenoid Groove *

**DOI:** 10.1055/s-0043-1767800

**Published:** 2023-10-06

**Authors:** Kelsey H. Mothersole, Seckin O. Ulualp, Romaine F. Johnson, Ashley F. Brown, Gopi B. Shah, Christopher C. Liu, Stephen R. Chorney

**Affiliations:** 1 Department of Otolaryngology, Head and Neck Surgery, University of Texas Southwestern Medical Center, Dallas, TX, United States; 2 Division of Pediatric Otolaryngology, Children's Medical Center, Dallas, TX, United States

**Keywords:** interarytenoid groove, injection laryngoplasty, children, dysphagia, endoscopic repair

## Abstract

**Introduction** Deep interarytenoid groove (DIG) may cause swallowing dysfunction in children; however, the management of DIG has not been established.

**Objective** We evaluated the subjective and objective outcomes of interarytenoid augmentation with injection in children with DIG.

**Methods** Consecutive children under 18 years of age who underwent injection laryngoplasty for DIG were reviewed. Data pertaining to demographics, past medical history, past surgical history, and results of pre and postoperative video fluoroscopic swallow study (VFSS) were obtained. The primary outcome measure was the presence of thin liquid aspiration or penetration on postoperative VFSS. The secondary outcome measure was caregiver-reported improvement of symptoms.

**Results** Twenty-seven patients had VFSS before and after interarytenoid augmentation with injection (IA). Twenty (70%) had thin liquid penetration and 12 (44%) had thin liquid aspiration before the IA. Thin liquid aspiration resolved in 9 children (45%) and persisted in 11 (55%). Of the 12 children who had thin liquid aspiration prior to IA, 6 (50%) had resolution of thin liquid aspiration after IA.

**Conclusions** Injection laryngoplasty is a safe tool to improve swallowing function in children with DIG. Further studies are needed to assess the long-term outcomes of IA and identify predictors of successful IA in children with DIG.

## Introduction

Laryngeal cleft is a rare congenital abnormality characterized by inadequate fusion of the interarytenoid tissue or cricoid cartilage. 1 2 3 The Benjamin-Inglis classification system groups clefts into four types based on depth. 4 Type I to type IV clefts represent increasingly severe communications extending from the interarytenoid region to the thoracic trachea. Palpation of the interarytenoid region is the gold standard to assess the extent of laryngeal cleft.

Type I laryngeal cleft is a supraglottic cleft that does not extend below the vocal folds. 4 5 6 7 8 9 10 11 12 13 The diagnosis of a type I laryngeal cleft requires meticulous examination of the interarytenoid region as it may be challenging to differentiate from a normal pediatric larynx. Clefts not reaching the true vocal folds have been described as a deep interarytenoid groove (DIG). 13 14 15 16 The clinical manifestations of a DIG are similar to those of type I laryngeal cleft. 13 While some cases may be asymptomatic, symptoms associated with clefts of varying interarytenoid mucosa height include dysphagia and respiratory abnormalities, such as chronic cough, stridor, respiratory distress, and aspiration pneumonia. Surgical management is considered for patients with persistent respiratory and feeding difficulty despite medical management and feeding therapy. 13

The definitive treatment for laryngeal cleft is endoscopic surgical repair; however, injection laryngoplasty has been increasingly used. To date, the surgical outcomes of the interarytenoid augmentation with injection (IA) have not been systematically studied. We hypothesized that injection laryngoplasty is an effective initial treatment trial for management of children with DIG. The primary objective of this study is to evaluate the subjective and objective outcomes of IA in children with DIG.

## Material and Methods

The electronic medical records of patients who had undergone IA for deep interarytenoid groove between January 2015 and February 2020 were reviewed retrospectively. The study was approved by the local institutional human research review board, and informed consent was waived. Patients younger than 18 years old who had videofluoroscopic swallow study (VFSS) before and after IA were included in the study. The exclusion criteria consisted of a history of abnormal vocal fold function, previous history of laryngeal cleft repair, prior IA, or history of airway surgery.

All patients underwent suspension laryngoscopy with palpation of the interarytenoid region for definitive diagnosis of DIG under general anesthesia. After placing laryngeal spreaders, the interarytenoid region was palpated using a using a right-angle laryngeal probe. The diagnosis of DIG was made when the interarytenoid groove did not extend to the level of the vocal fold. 13 14 15 16 The DIG height was not measured. Interarytenoid injection was performed using Prolaryn gel (aqueous/glycerin/carboxymethylcellulose gel - Merz North America, Raleigh, NC, USA) or Juvederm (hyaluronic acid - Allergan, Irvine, CA, USA). The interarytenoid area was injected until the groove was full. Patients who continued to have thin liquid penetration or aspiration after undergoing IA subsequently underwent DIG repair with endoscopic suturing.

Data pertaining to age, gender, race, past medical history, past surgical history, and results of pre and postoperative VFSS were obtained. The primary outcome measure was the presence of thin liquid aspiration or penetration on postoperative VFSS. Aspiration is defined as the passing of the bolus below the true vocal folds, and penetration is when the bolus enters the airway but not below the true vocal folds. 17 Secondary outcome measures included caregiver-reported subjective improvement of symptoms in the postoperative period. Comparisons of prevalence were performed by a chi-squared test. A *p* -value ˂ 0.05 was deemed statistically significant. Results included odds ratio (OR) with 95% confidence interval (CI).

## Results

Thirty-nine patients (22 male, 17 female, age range: 9 days–14 years) underwent IA. Thirty-six patients had no comorbid conditions. Comorbid conditions were gastroesophageal reflux disease in 10 patients, premature birth in 9, developmental delay in 7, asthma in 4, and genetic abnormality (two patients with Down syndrome, one with Duane syndrome, one with Trisomy 8, one with Emanuel syndrome and one with chromosome 4 abnormality) in 6 ( Table 1 ). The presenting symptoms were coughing or choking with feeds in 30 patients, aspiration pneumonia in 4, and recurrent upper respiratory infection in 2. The follow-up period ranged from 1 month to 11 months (median = 3). All patients had feeding therapy and modified consistency of feeds prior to IA. Patients with gastroesophageal reflux received antireflux therapy. Interarytenoid augmentation was achieved by injecting Prolaryn gel in 38 patients and Juvederm in 1. The amount of injection ranged from 0.1 ml to 0.2 ml (median = 0.1 ml). Three patients with no penetration or aspiration, as detected with VFSS, underwent IA due to clinical symptoms concerning aspiration. No surgical complications occurred. Twenty caregivers (51%) reported improved swallowing ( Table 1 ). Caregiver-reported swallowing assessment was unavailable in 10 patients. The rate of caregiver-reported improvement in girls was higher than in boys (OR: 3.39, 95% CI: 1.76–6.54, *p* < 0.001). Hispanic patients' caregivers reported higher rate of improvement in symptoms and caregivers of Caucasian (OR: 3.60, 95% CI: 1.79–7.23, *p* < 0.001) and African American patients (OR: 4.09, 95% CI: 2.05–8.18, *p* < 0.001). Caregivers of patients with genetic abnormality reported higher rate of improvement than those of patients with asthma (OR: 2.0, 95% CI: 1.05–3.80, *p* = 0.04) and developmental delay (OR:4.0, 95%CI:2.13–7.49, *p* < 0.001). The rate of caregiver-reported improvement in patients with gastroesophageal reflux disease was higher than patients with developmental delay (OR: 3.54, 95% CI: 1.91–6.55, *p* < 0.001) and premature birth history (OR: 2.36, 95% CI: 1.27–4.39, *p* = 0.009).

**Table 1:** Demographics of entire group of children with DIG who had injection and findings of caregiver reported symptoms

	*Overall, n (%)*	*Caregiver-reported* *improvement*	*Caregiver-reported* *no improvement*
***Gender***			
**Male**	22 (56)	10	7
Female*	17 (44)	10	2
***Race***			
**Caucasian**	24 (62)	12	7
Hispanic*	7 (18)	6	0
**African American**	7 (18)	3	2
**Asian**	1 (2)	0	0
***Comorbidity***			
** Asthma**	4 (11)	2	1
** Developmental delay**	7 (20)	2	2
** Gastroesophageal reflux disease**	10 (28)	7	2
** Genetic abnormality**	6 (17)	4	0
** Premature birth**	9 (24)	3	2
***Preop VFSS***	33 (85)	20	6
***Postop VFSS***	30 (77)	18	6

Abbreviations: N, number; VFSS, videofluoroscopic swallow study.

**Fig. 1:**
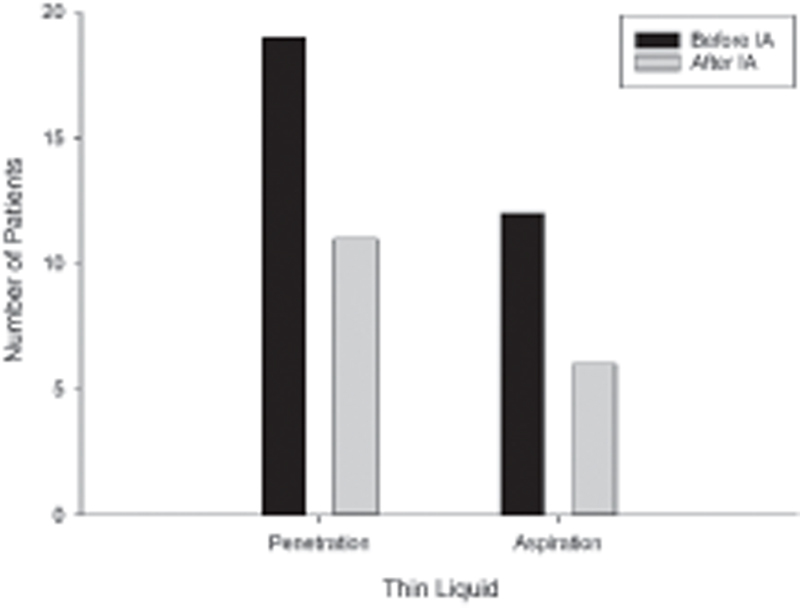
Number of patients with thin liquid penetration and aspiration before and after interarytenoid augmentation with injection (IA).

Twenty-seven patients (17 male, 10 female, age range: 4 months–7 years) had VFSS before and after IA ( Table 2 ). Videofluoroscopic swallow study was not available in six patients prior to IA and in seven patients after IA. Furthermore, it was obtained between 1 and 3 months after IA in 25 patients. Two patients had VFSS 1 week and 6 months after IA. Of the 27 children who had VFSS before and after IA, 19 (70%) had thin liquid penetration and 12 (44%) had thin liquid aspiration before IA ( Fig. 1 ). Thin liquid penetration resolved in 9 children (47%) and persisted in 10 (53%). Six of the 8 children (75%) who had no thin liquid penetration before IA developed thin liquid penetration after IA. Of the 12 children who had thin liquid aspiration prior to IA, 6 (50%) had resolution of thin liquid aspiration after IA. After IA, thin liquid penetration occurred less in male (OR: 2.03, 95% CI: 1.15–3.59, *p* = 0.02) and Hispanic patients (OR: 4.89, 95% CI: 2.66–8.97, *p* < 0.001), and caregivers reported improvement of symptoms (OR: 2.3, 95% CI: 1.34–4.22, *p* = 0.004). After IA, thin aspiration occurred less in patients older than 1 year (OR: 2.03, 95% CI: 1.14–3.59, *p* = 0.02), Caucasian (OR: 2.57, 95% CI: 1.45–4.56, *p* = 0.002), and those with presence of comorbidity (OR: 4.0, 95% CI: 2.2–7.2, *p* < 0.001).

**Table 2:** Findings of thin liquid penetration and aspiration before and after interarytenoid augmentation with injection

						Thin liquid penetration		Thin liquid aspiration	
**Subject**	**Age**	**Gender**	**Race**	**Comorbidity**	**Caregiver-reported improvement**	**Before**	**After**	**Before**	**After**
**1**	1	F	AA	No	Yes	+	+	−	−
**2**	3	M	C	Yes	Not available	+	+	+	−
**3**	2	F	C	Yes	Yes	−	+	−	+
**4**	10mo	F	C	No	Yes	+	−	+	+
**5**	2	F	AA	Yes	Yes	+	−	−	−
**6**	8mo	M	C	No	No	+	+	+	+
**7**	3	M	C	Yes	Yes	+	+	−	−
**8**	2	M	C	No	Not available	+	+	+	−
**9**	8mo	M	H	Yes	Yes	+	+	−	−
**10**	2	F	H	Yes	Not available	−	+	+	−
**11**	2	F	C	Yes	No	+	+	−	−
**12**	4	M	C	Yes	Yes	+	−	−	−
**13**	2	M	H	No	Yes	+	−	+	+
**14**	**7**	**M**	**C**	**Yes**	**Yes**	**+**	**+**	**-**	−
**15**	2	F	C	Yes	No	−	−	−	−
**16**	9mo	M	H	Yes	Yes	+	−	+	−
**17**	11mo	M	H	Yes	Yes	−	+	−	−
**18**	11mo	F	C	No	Yes	+	+	+	+
**19**	1	M	C	Yes	No	+	−	−	−
**20**	2	M	AA	Yes	No	−	−	+	+
**21**	17mo	M	C	Yes	Yes	−	+	−	−
**22**	4	M	C	Yes	Yes	+	−	+	−
**23**	18mo	F	H	No	Yes	+	−	−	−
**24**	4mo	M	AA	Yes	Yes	−	+	+	+
**25**	5	M	C	No	Yes	+	−	−	+
**26**	1	F	C	No	Yes	−	+	+	−
**27**	18mo	M	C	Yes	Not available	+	+	−	−

Abbreviations: AA, African American; C, Caucasian; F, female; H, Hispanic; M, male; mo, month; +, present; -, absent.

Endoscopic repair was performed in eight children who had IA ( Table 3 ). Interarytenoid augmentation with injection has resulted in resolution of thin liquid penetration or aspiration in five children, and no change in three. After endoscopic repair, the child with worsening symptoms after IA (subject 21) had resolution of thin liquid penetration. Of the 4 children (subjects 6, 9,15, and 16) with no change in penetration or aspiration after IA, 1 had resolution of penetration and aspiration and 3 had no change. Three of the 6 the children who had no change or worsening after IA had no thin penetration after endoscopic repair. A child with resolution of thin liquid penetration after IA (subject 5) continued to have no thin liquid penetration after endoscopic repair.

**Table 3:** Findings of thin liquid penetration and aspiration before and after interarytenoid augmentation with surgery

						Thin liquid penetration		Thin liquid aspiration	
**Subject**	**Age**	**Gender**	**Race**	**Comorbidity**	**Caregiver-reported improvement**	**Before**	**After**	**Before**	**After**
**5**	2	F	AA	Yes	Not available	+	−	−	−
**6**	8mo	M	C	No	No	+	+	+	+
**8**	2	M	C	No	Yes	+	−	+	−
**9**	8mo	M	H	Yes	No	+	+	−	−
**14**	7	M	C	Yes	Yes	+	+	−	−
**16**	9mo	M	H	Yes	Yes	+	−	+	−
**21**	17mo	M	C	Yes	Yes	+	−	−	−
**24**	4mo	M	AA	Yes	Yes	+	−	+	−

Abbreviations: -, absent; +, present; AA, African American; C, Caucasian; F, female; H, Hispanic; M, male; mo, month.

## Discussion

Interarytenoid augmentation with injection is an attractive option used to diagnose and treat type I laryngeal cleft. In the present study, IA was used to treat swallowing dysfunction in children with DIG. Subjective and objective improvement of swallowing function was documented in 47 to 51% of the children who had IA. The rate of caregiver-reported improvement in swallowing function (51%) was similar to the resolution rate of thin liquid aspiration (50%). The resolution rate of thin liquid penetration was similar to that of thin liquid aspiration. Plausible explanations of persistent aspiration and penetration after IA include inadequate augmentation of the interarytenoid groove and presence of comorbidities such as neuromuscular and developmental abnormalities affecting swallowing function. 18 19

The outcomes of IA for the management of type I laryngeal cleft have been evaluated based on resolution or improvement of penetration or aspiration as detected with VFSS. 18 19 20 21 22 23 The resolution rate of aspiration after IA ranged from 13 to 56%. 18 21 22 The long-term follow-up revealed resolution of aspiration in 37% of children. 19 Clinical improvement of aspiration and penetration based on ability to use a less thick consistency or resolution of aspiration ranged from 48 to 57%. 20 23 In the present study, the rates of resolution of penetration (47%) and aspiration (50%) in children with DIG were comparable to previous success rates in children with type I laryngeal cleft. 21 22 The depth of the interarytenoid groove as well as differences in patient demographics and morbidities may account for higher success rate in children with DIG than that of a previous study in children with type I laryngeal cleft. 18

A wide variety of injection materials such as hyaluronic acid, autologous fat, carboxymethycellulose gel, micronized acellular dermal matrix, and calcium hydroxylapatite is used for injection laryngoplasty. 24 Gelfoam, calcium hydroxylapatite, aqueous/glycerin/carboxymethylcellulose gel or carboxymethylcellulose gel for interarytenoid augmentation have been used in children with type I laryngeal cleft. 18 19 20 21 22 23 24 25 In the present study, aqueous/glycerin/carboxymethylcellulose gel was used in all children with DIG except one patient who received hyaluronic acid. The resorption of carboxymethylcellulose gel usually occurs within 3 to 6 months after injection per Prolaryn gel instructions for use; however, other injection materials are absorbed in between 3 to 12 months, depending on the material. 26 The effect of varying laryngeal injection materials on the outcomes of IA is unknown in children with DIG or type I laryngeal cleft.

Worsening of dysphagia after IA has been documented in children with type I laryngeal cleft. 20 The exact mechanism of worsening of dysphagia after IA is unknown. Patient-dependent factors such as age, comorbidities, timing of swallowing assessment after surgery, and variations in surgical technique may have contributed to deterioration of the swallowing function. 20 Videofluoroscopic swallow study is a moment in time and provides a snapshot of the swallowing function. Its results are influenced by fatigue, bolus volume, variability from day to day or over the course of a day, cooperation, variability in feeders, and drinking utensil differences. 27 28 The effect of VFSS-dependent factors on findings worsening dysphagia cannot be excluded. Endoscopic repair resulted in resolution of thin liquid penetration in 3 of the 6 the children who had no change or worsening after IA. Hence, our findings provide preliminary evidence to support the use of endoscopic repair in children with DIG who do not benefit from IA.

The management of children with DIG has not been established. The management of algorithms for type I laryngeal cleft recommended arytenoid augmentation by injection or suturing after failure of medical management. 16 22 Interarytenoid augmentation with injection was proposed as an intermediary step at the time of type I laryngeal cleft diagnosis. 20 In the present study, IA was performed after patients received feeding therapy and medical treatment. Conceivably, IA may be performed at the time of diagnosis or failure of medical management in children with DIG. The limitations of the present study are inherent to the retrospective study design. Interarytenoid augmentation with injection was performed by multiple surgeons, and the fullness of the interarytenoid area after injection was determined based on surgeon's judgement. The effect of possible variations in postinjection interarytenoid groove fullness on present study findings cannot be excluded. The volume of injectate was not available in all patients; however, the volume of injectate varies depending on the height of interarytenoid groove. Therefore, the interarytenoid area was injected until the groove was full as described in previous studies, achieving fullness of the groove provided uniformity amongst the patients. 21 23 The severity of penetration and aspiration was not assessed using a standard scale. The use of a standard scale would facilitate interstudy comparisons and provide better characterization of dysphagia. Preoperative VFSS was not available in six patients; however, we recommend objective assessment of swallowing function with the use of pre and postoperative swallow study. Pre and postoperative VFSS is crucial to determine the outcomes of surgical intervention.

The definition of DIG has been debated amongst members of the International Pediatric Otolaryngology Group. 13 The majority of members (85%) made the DIG diagnosis based on visual inspection of the interarytenoid groove. The DIG was described as an interytenoid groove approaching but not reaching the level of the true vocal folds. 13 Few members (15%) made the DIG diagnosis when the microscopically measured interarytenoid groove height was less than 3 mm and the interarytenoid groove remained above the true vocal fold. In the present study, the diagnosis of DIG was made when the palpation of the interarytenoid groove revealed a cleft not extending to the level of the vocal fold. As there is no universally accepted gold standard method to diagnose DIG, the clinical features of a patient with DIG should be considered in its management. The identification of an objective criterion to define DIG resulting in clinical symptoms merits further investigation. The disproportionate representation of subgroups of age, gender, race, and comorbidity may influence the outcomes of comparisons; therefore, our results regarding the rate of caregiver reported improvement and resolution of thin liquid penetration and aspiration amongst the subgroups of gender, age, race, and comorbidity should be interpreted carefully.

## Conclusion

Injection laryngoplasty is a safe tool to improve the swallowing function in children with DIG. Interarytenoid augmentation with injection improved thin liquid aspiration and penetration; however, worsening of dysphagia may occur after IA. Endoscopic repair improved the swallowing function in children who had no benefit from IA or worsening of thin liquid penetration after IA. The assessment of long-term outcomes of injection laryngoplasty and the identification of predictors of success after injection laryngoplasty in children with DIG merit further investigation.
